# The Adult Difficult Intravenous Access (DIVA) Cognitive Aid: An Evidence-Based Cognitive Aid Prototype for Difficult Peripheral Venous Access

**DOI:** 10.7759/cureus.37135

**Published:** 2023-04-04

**Authors:** Philip L Stagg

**Affiliations:** 1 Faculty of Health Sciences and Medicine, Bond University, Gold Coast, AUS

**Keywords:** ultrasound-guided blocks and vascular access, seldinger technique, heuristics and biases, fixation error, choice architecture, behavioural psychology, patient safety, cognitive aids, difficult intravenous access

## Abstract

Difficult intravenous access (DIVA) is common, with imperfect solutions. Cognitive aids are widespread in anaesthesia; however, a standard DIVA cognitive aid is lacking. This article describes a cognitive aid for DIVA. It has been developed using evidence-based techniques for DIVA. The effects of heuristics, biases, and automatic thinking on procedural decision-making are briefly discussed. While often useful, shortcut decision-making can impair the performance of apparently simple tasks. Cognitive aids may lead to better outcomes by providing choice architecture. This resource is intended as a cognitive aid prototype for difficult peripheral venous access, incorporating both modern behavioural psychology principles and evidence-based medicine. It may be used as both an educational tool, or as a cognitive aid to assist in situations where DIVA is encountered or expected. The adult DIVA cognitive aid is intended for use in both elective and emergency scenarios by practitioners adequately trained in ultrasound-guided or ultrasound-assisted vascular access and Seldinger-based techniques. Clinical implementation and audit of the adult DIVA cognitive aid, or similar locally developed cognitive aids based on this prototype are recommended.

## Introduction

Intravenous (IV) access is the most common invasive hospital procedure, being undertaken in up to 80% of hospitalised patients [1]. Unfortunately, difficult IV access (DIVA) is common, occurring in up to 39% of patients [2]. DIVA can be defined as two or more failed attempts at IV access [3] or the requirement of a more experienced care provider [2]. Witting et al. Found that DIVA resulted in treatment delays of up to 120 mins in some cases [2]. IV access is painful for patients [2]. The A-DIVA scale, a prediction tool for the identification of DIVA in adults, identified 5 factors predictive of DIVA: a history of DIVA, difficult peripheral venous access anticipated by the practitioner, veins that are neither palpable nor visible, and a vein diameter less than 3 millimetres after tourniquet application [4]. Prediction of DIVA is clinically useful, as it has the potential to not only identify situations where treatment delays may occur, but it allows the team to consider contingency plans, such as the need for more experienced practitioners, advanced equipment such as ultrasound, in the case of time-critical emergencies, early preparation of central venous or intraosseous (IO) access.

In general, the use of cognitive aids is now common in clinical practice. Their potential utility is highlighted by studies that have demonstrated that the recall of memorised clinical algorithms is poor during a crisis [5,6]. Stress and fixation errors are significant contributors to error [7,8]. Cognitive aids can reduce human error and have been shown to significantly increase adherence to crisis management algorithms and significantly reduce missed steps [5,6]. The World Health Organisation (WHO) Surgical Safety Checklist reduced post-operative complications and mortality by approximately 36% [9].

Heuristics, biases, and automatic thinking affect decision-making. While often useful, shortcut decision-making (heuristics) can impair the performance of even apparently simple tasks [10]. Fixation errors and automatic actions have contributed to deleterious outcomes [11]. Such cognitive blind spots dictate that cognitive aids for seemingly easy yet error-prone tasks such as IV cannulation could lead to improved outcomes.

This manuscript presents the adult DIVA cognitive aid, a cognitive aid proposal that has been designed to address these issues. It is intended to serve as a foundation teaching tool and a cognitive aid when performing cannulation in the context of DIVA. While primarily intended for use by critical care practitioners in elective and emergency scenarios, it may be suitable for a broad range of clinical environments where DIVA is encountered or utilised and anyone who has been adequately trained in ultrasound-guided or ultrasound-assisted vascular access and the Seldinger technique. As such, it may be potentially useful for clinicians over a broad range of abilities.

The adult DIVA cognitive aid has been developed using evidence-based techniques for DIVA as well as behavioural psychology concepts such as choice architecture. It is hoped that it may improve outcomes for patients and staff alike, in terms of success rates and patient satisfaction. It is presented here as a proposal or prototype for the future development of DIVA cognitive aids, as a much-needed clinical resource.

## Technical report

The DIVA cognitive aid

**Figure 1:**
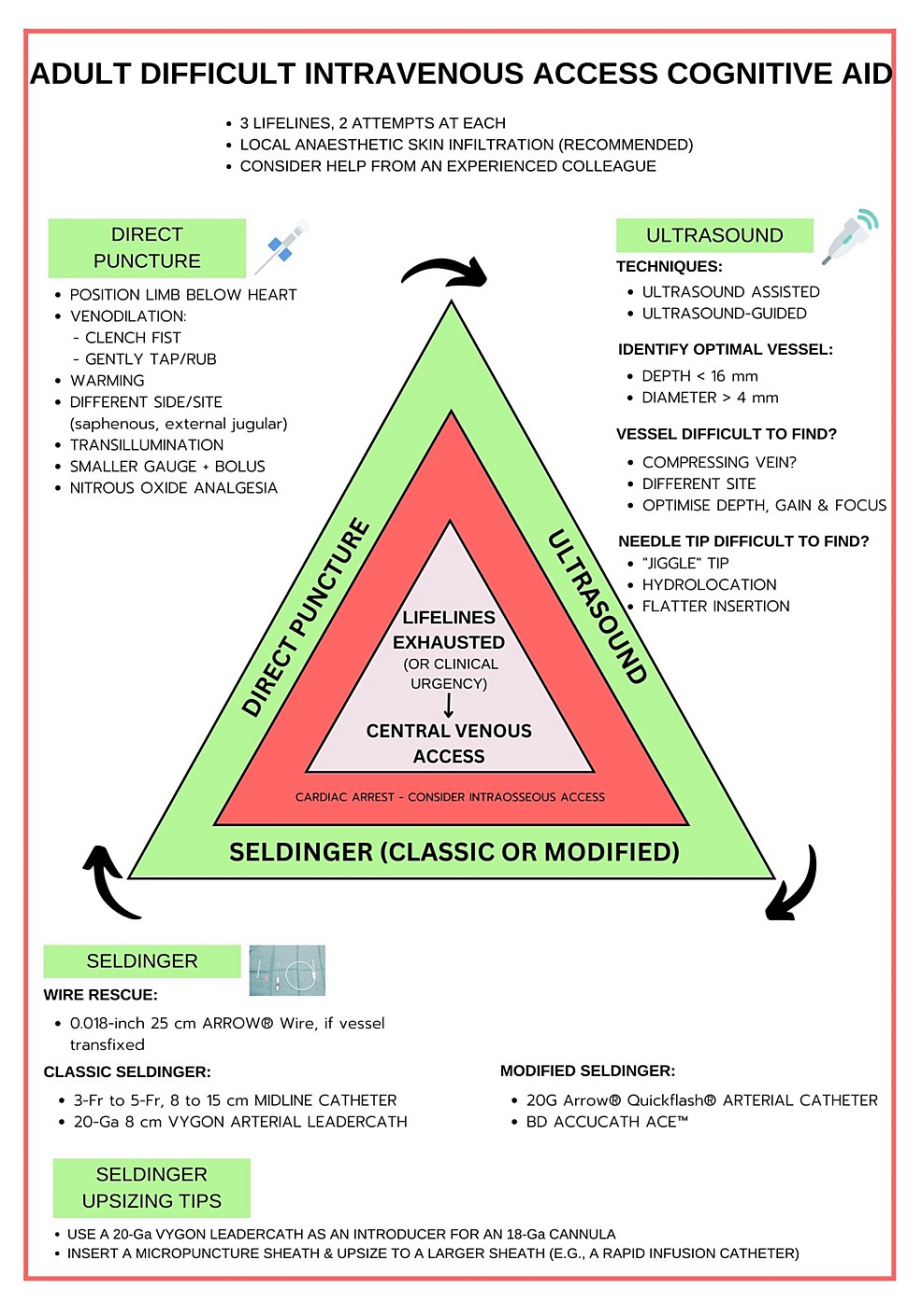
The adult difficult intravenous access (DIVA) cognitive aid: a cognitive aid and educational resource for difficult peripheral venous access The DIVA cognitive aid consists of three vascular access ‘lifelines’ within the green (safe) zone: (i) Direct Puncture, (ii) Ultrasound, and (iii) Seldinger lifelines. While the sequence of lifeline utilisation is not fixed, starting with direct puncture, and progressing to ultrasound-based techniques (real-time guidance or ultrasound-assisted) and Seldinger-based techniques is recommended. A maximum of two attempts are permitted at each lifeline before progressing to the next. If all lifeline attempts are exhausted, or the patients clinical condition deteriorates, practitioners are prompted to move to the central zone, where they perform central venous or intraosseous access as dictated by the clinical scenario. In a time-critical emergency, practitioners proceed directly to the central zone and perform emergency access for resuscitation. At least one vascular access attempt should be performed by an alternative experienced practitioner, when available, before utilising central zone techniques. Seldinger upsizing tips are provided for circumstances where wide-bore access solutions are required.

**Figure 2:**
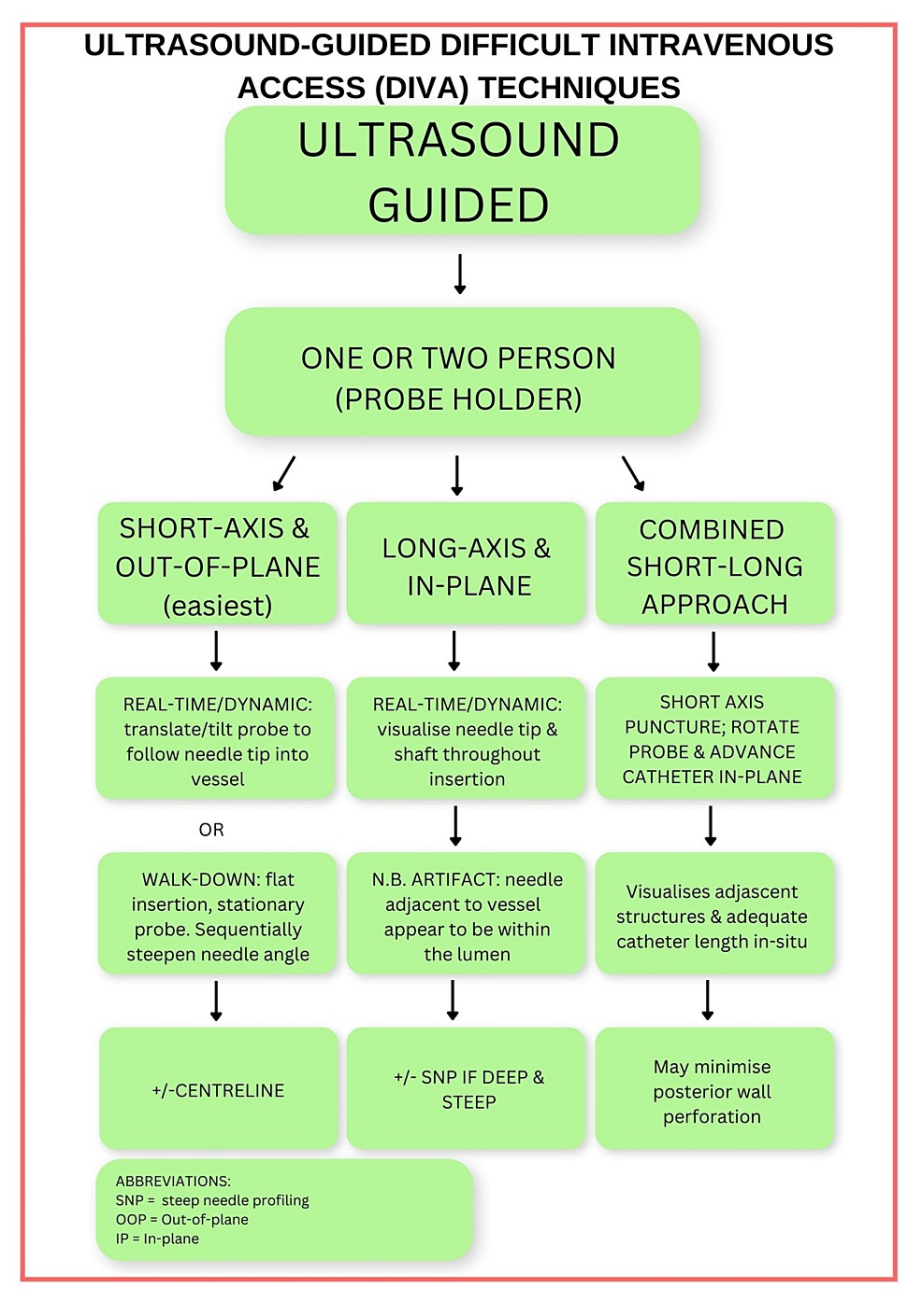
Real-time ultrasound-guided difficult intravenous access (DIVA) techniques. Three techniques for ultrasound-guided peripheral venous access are outlined, including the short-axis, out-of-plane approach, the long-axis, in-plane approach, and the combined short-long approach. A one- or two-person technique can be employed, with a second person acting as a probe holder in the two-person technique. Machine aids such as the centreline function, or steep needle profiling (SNP) may assist in improving accuracy and needle visualisation. Dynamic/real-time ultrasound-guided techniques, where the needle tip is tracked under constant vision into the target vessel are recommended, as they have the strongest evidence base. The exact technique employed can be determined by user preference and training.

**Figure 3:**
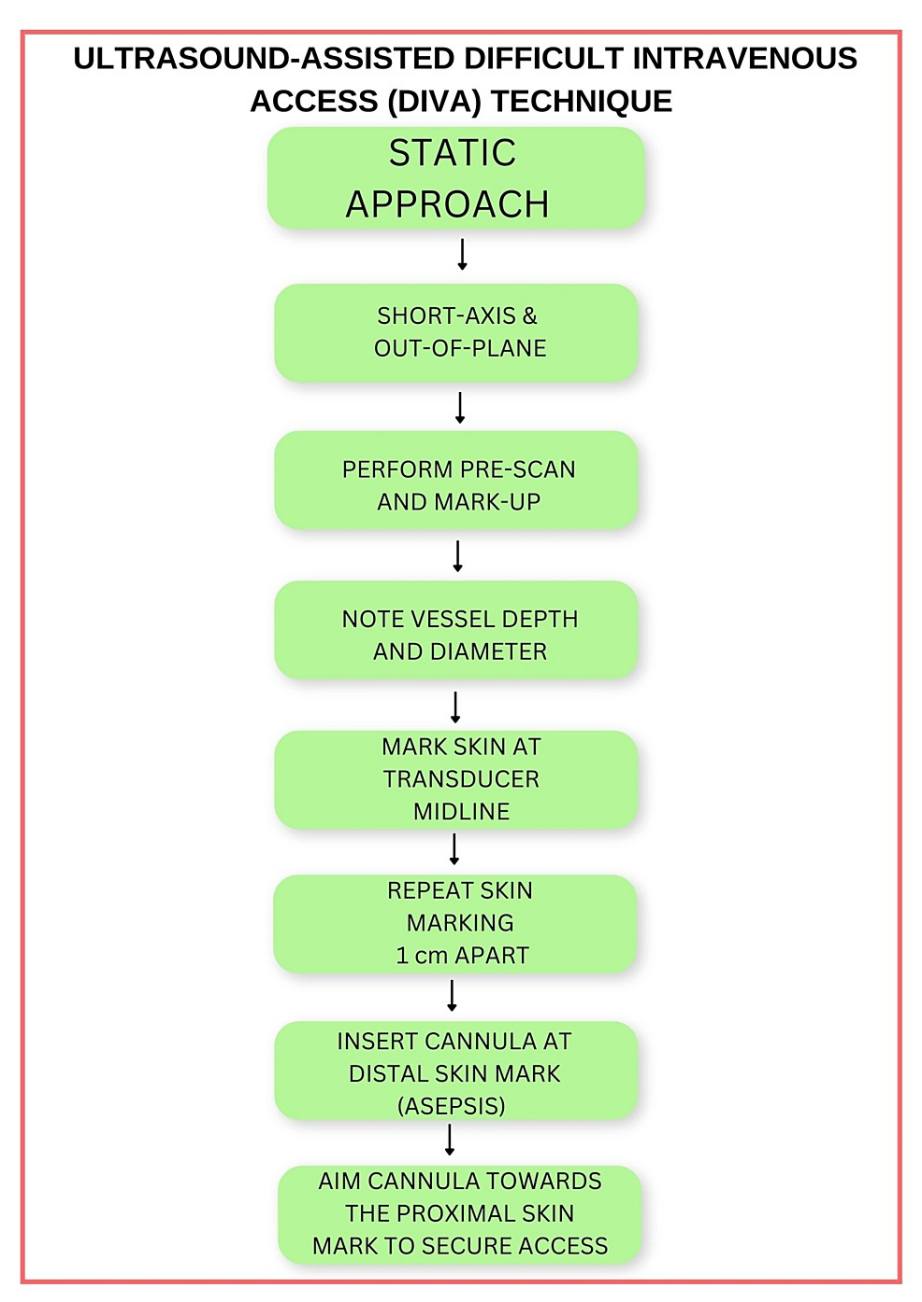
Static ultrasound-assisted difficult intravenous access (DIVA) technique. A potential technique for static ultrasound-assisted peripheral venous cannulation is outlined, whereby ultrasound is utilised to mark the skin at two points, 1 cm apart, overlying the target vessel. The skin is there and punctured at the distal point while aiming the cannula towards the proximal point, until vascular access is achieved. This technique suits less experienced practitioners not yet competent in ultrasound-guided approaches.

The Vortex Approach is a popular cognitive aid and methodology for emergency airway management [12]. The DIVA cognitive aid (Figure 1) is modelled after the Vortex implementation tool and contains similar terms and concepts for consistency and familiarity. Such qualities are considered desirable in high-fidelity tasks [12]. In addition to the primary cognitive aid, two supplementary cognitive aids provide instruction on real-time ultrasound-guided, and static ultrasound-assisted cannulation (Figures 2, 3).

Indications 

**Figure 4:**
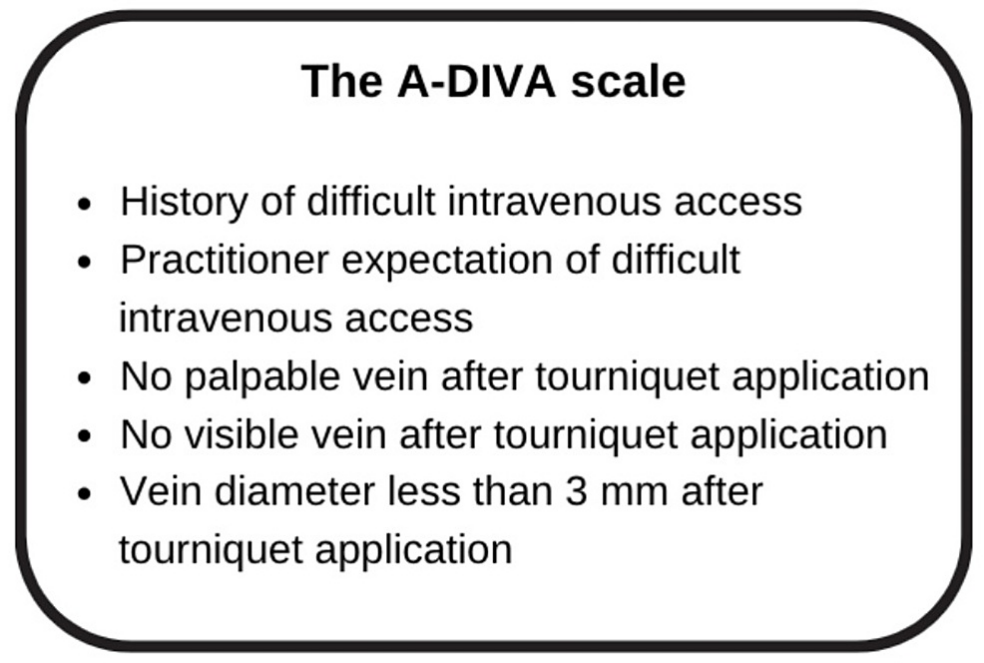
The A-DIVA scale The A-DIVA scale, adapted from [4]. van Loon et al. determined via multivariate regression analysis that each factor is significantly associated with DIVA, with two risk factors being associated with a 37% failed first attempt at cannulation, rising to 98% first-attempt failure with five risk factors.

The adult DIVA cognitive aid is prospectively indicated in patients with predictors of DIVA as defined in the modified A-DIVA scale published by van Loon et al. [4], or after two failed attempts at conventional direct-puncture cannulation after which DIVA is established [2]. The A-DIVA scale used to predict DIVA is shown in Figure 4.

Lifelines

In keeping with the Vortex cognitive aid, the adult DIVA cognitive aid consists of three vascular access ‘lifelines’: (i) Direct Puncture, (ii) Ultrasound, and (iii) Seldinger lifelines.

Using this paradigm, the practitioner performs two attempts at a vascular access lifeline, before moving on to the next lifeline. The sequence of lifeline utilisation is not fixed, in keeping with the Vortex model; however, the natural progression in most instances would be to commence with the direct puncture lifeline. Local anaesthetic skin infiltration with a 25-Ga (or smaller) insulin syringe is recommended and has been shown to reduce pain for adult cannulation for cannula gauges 22-Ga or larger [13]. The adult DIVA cognitive aid prompts practitioners to enlist help, with at least one attempt to be performed by an additional experienced practitioner where feasible.

The green zone 

The green zone indicates that all is well while the patient condition is stable, and cannulation attempts are limited to two per lifeline. However, after a maximum of six attempts at cannulation (two at each lifeline), or earlier if dictated by clinical urgency (e.g., patient deterioration or haemodynamic instability) practitioners progress into the central zone, and central venous access is warranted. In rare circumstances, such as a cardiac arrest scenario in the emergency department, IO access may be warranted.

Direct puncture lifeline

Direct puncture is the usual approach to peripheral IV cannulation; however, several basic optimisations may improve success. Lowering the limb below the level of the heart [14], fist clenching (vasodilatory chemical mediators, venous pumps) [15], gentle rubbing or tapping on the cannulation site [16], milking blood from proximal to distal [16], and limb warming [17], are simple manoeuvres that can increase venous prominence. If visible or palpable veins are not forthcoming, examining additional sites are recommended [16]. Useful sites include the long saphenous, external jugular, or anterior wrist vessels. These sites are often overlooked. Transillumination may be useful [18]. Starting with the smallest possible vessel or cannula can be beneficial, either for the induction of anaesthesia, or for systemic fluid loading, or by infusion of 100 mL of crystalloid into a small vein with a tourniquet applied to engorge the veins of that limb. Vascular access may then improve, and a larger cannula can be inserted [19]. Nitrous oxide can reduce patient distress and increase compliance [20]. This adjunct may be useful, if available, for anxious patients.

Ultrasound lifeline

Van Loon et al. conducted a systematic review and meta-analysis including eight studies and 1,660 patients and found that ultrasound guidance significantly increased cannulation success rates, particularly for DIVA, with an odds ratio of 2.49 (95% confidence interval 1.37-4.52, P=0.003) [21]. In addition, ultrasound guidance reduced the number of cannulation attempts, reduced time, and improved patient satisfaction, but without a reduction in complications [21]. International consensus guidelines recommend ultrasound guidance when difficult peripheral venous access is anticipated or encountered [22,23]. Detailed reviews of ultrasound-guided cannulation techniques are available [24,25]. Optimal target vessels for ultrasound-guided cannulation are those with a diameter greater than > greater than 4 mm, and at a depth between 3 mm and 15 mm [26].

The adult DIVA cognitive aid prompts static ultrasound-assisted techniques such as pre-mapping and skin mark-up (e.g., less experienced practitioners) or real-time ultrasound-guided techniques. Further delineation of these techniques is provided to users in Figures 2, 3.

When an appropriate target vessel is difficult to find, practitioners should examine an alternate limb or site [16,25]. Ensure that the target vessels are not compressed due to excessive probe pressure [25]. Optimising depth, gain, and focus on the ultrasound machine will help improve vessel and needle visualisation [27].

Real-time needle-tip visualisation can be further improved by hydro-location [28], such as using a local anaesthetic ‘seeker needle’ and injecting a small amount of local anaesthetic, and tissue movement created by jiggling the needle tip [29]. Checking for correct needle-probe alignment and ensuring that the needle approach to the skin is relatively flat, to ensure the greatest number of echos reflect off the needle and return to the transducer, thus improving image quality [30]. 

Figure 2 outlines real-time ultrasound-guided approaches to DIVA where additional prompts are required. A one- or two-person technique can be used, with the assistant holding the probe if required [31].

Ultrasound-guided approaches include out-of-plane (vessel in short axis), in-plane (vessel in long axis), or the combined “short-long axis approach”. Short-axis and long-axis ultrasound-guided techniques are well described [24,25]. Compared to the in-plane approach, the out-of-plane approach is more widely used, has a higher success rate, allows for better visualisation of the surrounding structures, and is easier to learn [24,25,32,33]. However, it is associated with a higher incidence of posterior wall perforation, and distinguishing the needle tip from the needle shaft on the ultrasound image can be difficult [24,25,33]. By comparison, the in-plane approach allows visualisation of both the needle tip and shaft from initial skin puncture through to vessel cannulation. It allows real-time visualisation of the catheter being advanced unencumbered over the needle and into the vessel, and the determination of whether an adequate length of catheter remains within the vessel lumen upon procedure completion. Bahl et al. found that greater than 2.75 cm of catheter should dwell within the vessel to optimise catheter dwell time and reduce catheter dislodgement [34]. Unfortunately, side-lobe artifact is common with in-plane ultrasound-guided cannulation. This artifact occurs when the cannula appears to be within the vessel lumen when it is actually adjacent to it [24]. Lamperti et al. recommend proficiency in both in-plane and out-of-plane techniques [22]. A combined short-long axis technique may be useful [35,36]. Initial venipuncture is achieved with the needle tip out-of-plane and the vessel in the short axis. The transducer is then rotated 90˚ such that the needle is in-plane and the vessel in the long axis as the cannula is advanced. Compared to the short-axis approach, the combined approach significantly increased central venous access success, with reduced vessel posterior wall perforation in adults and neonates [35,36]. However, while these studies allude to potential benefits of a combined short-long approach for peripheral venous access, direct evidence of benefit when this technique is used for DIVA is lacking.

When using the short-axis technique for ultrasound-guided cannulation, Figure 2 guides users to consider using dynamic real-time techniques, such as dynamic needle-tip visualisation (DNTV) [24,25,37,38] or the walk-down technique [39]. Dynamic techniques can refer to any real-time technique for detecting direct or indirect signs of vessel puncture. Randomised trials have demonstrated that dynamic techniques have superior performance compared to both direct puncture cannulation [37], and static ultrasound-assisted vascular access [40], although they require higher technical proficiency [22,24,25,40]. Guidelines recommend a real-time ultrasound-guided technique where possible [22,25].

Dynamic needle-tip visualisation involves translating, tilting, or fanning the ultrasound probe as the needle is advanced in the out-of-plane view, to keep the needle tip in view [24,25,37,38]. The “target sign” is a helpful sign to determine that the needle tip has entered the lumen of the vessel [38]. The target sign is typified by the white, hyperechoic needle tip appearing in the centre of the black, hypoechoic vessel. Whilst DNTV is one of the most recommended real-time techniques for ultrasound-guided cannulation, it is an advanced skill due to the requirement for good hand-eye-coordination skills. A further challenge with this technique is determining whether the white hyperechoic dot at the centre of the target is the needle tip or the needle shaft. The “vanishing target sign” can help in this regard, whereby the ultrasound beam is fanned distally to see the target sign (the white hyperechoic dot) appear to vanish whilst still in the middle of the vessel lumen, providing confirmation that the needle tip is indeed within the lumen [41].

The walk-down technique entails keeping the transducer stationary while the needle is incrementally inserted and withdrawn to just under the skin surface, successively angling the needle steeper until the target is reached [39]. The initial insertion is initially shallow (almost flat), to facilitate needle-tip visualisation, given that needle echogenicity is greatest when needle-beam alignment is closest to perpendicular [30]. This technique derives from regional anaesthesia and suits a single-operator technique. Less hand-eye coordination is required, as the ultrasound transducer does not need to be translated or fanned to keep the needle tip in view; however, a greater number of needle passes are required to get the trajectory correct. This technique would potentially suit practitioners with intermediate ultrasound-guided cannulation ability, due to the reduced coordination requirement with a stationary probe, while still harnessing the benefits of a real-time technique.

While guidelines recommend an ultrasound-guided technique for DIVA, a static ultrasound-assisted technique is still considered useful, particularly when operator experience is low [22,38,40,42]. A description of static ultrasound-assisted approach to DIVA is included in Figure 3. Bain et al. demonstrated in a randomised controlled trial that ultrasound-assisted cannulation increased success rate, reduced procedure time, and the number of needle redirections in children with DIVA [42]. Two skin markings, 1 cm apart are recommended [42]. After marking the skin overlying the target vessel with a surgical marking pen using ultrasound assistance, the skin is cleaned, and the cannula at the distal mark. The cannula is there an advanced towards the proximal mark until vascular access is achieved. Of note, it is important to immediately attempt cannula insertion after marking the skin, as the skin-markings may not overlie the vessel if the patient changes position [38]. Advantages of the ultrasound-assisted technique over conventional cannulation include the acquisition of information such as vessel depth, location, calibre, the presence or absence of thrombus, the vessels relationship to surrounding structures, and the absence of vein compression by the probe during cannulation attempts [38,42]. It is most useful for less experienced practitioners, as the advanced hand-eye coordination and sonography skills required to keep the needle tip in view during real-time ultrasound-guided cannulation are not necessary. However, when possible, a real-time approach is recommended [22,25,37,38,40].

Machine aids can be useful when needle visualisation is poor during ultrasound-guided cannulation [27]. The centreline function is useful for the short-axis/out-of-plane approach. An electronic beam steering function or SNP on newer machines can be helpful for the long-axis/in-plane technique [27]. A manual ‘heel-toe’ manoeuvre can also help optimise needle-beam alignment and echogenicity [27]. Although useful, these are not included in the DIVA cognitive aid, in the interest of space and clarity. Similarly, advanced ultrasound technologies such as needle guides, laser guides, 3D/4D ultrasound, and robotics [43] are not included in the adult DIVA cognitive aid as they are not routinely available.

Seldinger lifeline 

Both the classic- and modified-Seldinger techniques have been shown to improve vascular access success [44]. The Arrow® 0.018 inch (0.46 mm) 25 cm spring-wire guide (Teleflex®, Wayne, PA, USA) can be used to rescue a cannula after inadvertently transfixing a vessel during direct-puncture or ultrasound-guided peripheral venous cannulation. Traditionally this technique, known as the “through-and-through" or “transfixation” technique, was used for arterial cannulation where both anterior and posterior vessel walls were intentionally punctured. After transfixation, the cannula was withdrawn until flashback, a wire advanced into the vessel and the cannula then advanced over the wire. For arterial cannulation, intentional transfixation has been shown to be inferior compared to single wall puncture [45]. Nonetheless, it can still be useful as a rescue technique for both venous and arterial cannulation after accidental posterior wall puncture. However, extravasation injury due to fluid leakage can potentially occur with vessel damage or perforation [23], so a higher index of suspicion is warranted if this rescue technique is utilised.

The 20-Ga (3-Fr) 8 cm and 18-Ga (4-Fr) 10 cm Vygon Arterial Leadercath (Vygon, Écouen, France) is the most common repurposed device for DIVA [46,47]. Alternatively, the Vygon Arterial Leadercath can also be used as a coaxial introducer to facilitate insertion of 18-Ga and 16-Ga long cannulas, as a 48 mm 18-Ga BD Insyte catheter (Becton Dickinson, Utah, USA) fits seamlessly over a 20-Ga Vygon Leadercath, and a 16-Ga Insyte catheter similarly fits seamlessly over an 18-Ga Vygon Leadercath [48]. This circumvents the need to use the Vygon Leadercath as a peripheral venous cannula, which is not an optimal venous cannula with its red-coloured hilt, longer length, and given that it has not been specifically designed for fluid and drug administration. Utilising the Vygon Leadercath as a coaxial introducer for larger-bore cannula, however, harnesses the insertion benefits of this common device, and provides an easily cannula upsizing technique.

Midline catheters are useful for DIVA and afford long dwell times with minimal complications [24,27,49]. They are useful for deep veins, as a greater length of catheter within the vessel minimises catheter failure [24,49]. Midline catheters range from 3-Fr to 5-Fr and a length of 8 cm to 25 cm and are pressure-rated at 300 psi. The echogenic introducer needle is useful for DIVA.

Devices with integral wires include the AccuCath Ace™ (Becton Dickinson) and the Arrow® Quickflash® Arterial Catheter (Teleflex, Wayne, PA, USA). These devices have demonstrated good results for ultrasound-guided cannulation [50,51].

Micropuncture kits including the 4-Fr to 5-Fr 10 cm Micropuncture® Access Set (Cook Medical, Bloomington, IN, USA) and V•Stick™ Vascular Access Set (Argon Medical, Plano, TX) are useful for DIVA [52], and offer the added benefit that they can be serially upsized to wide-bore sheaths, including a rapid infusion catheter (RIC®) (Arrow® International, Morrisville, NC, USA) [53]. They are purpose-designed to accommodate a 0.038-inch (0.89 mm) wire, allowing a range of coaxial-dilator-mounted percutaneous sheaths to be introduced. Micropuncture kits also contain an echogenic needle.

Central zone: time-critical emergency, lifelines exhausted, or clinical deterioration

The centre of the DIVA cognitive aid primes practitioners to obtain urgent central venous (or IO access) after exhausting all lifelines, if there is a deterioration in the patient’s condition, or in time critical emergencies. The concept of priming is fundamental to modern airway management guidelines [12,54], and is intended to accelerate clinical progression and minimise barriers to performing more complicated, clinically daunting, or otherwise undesirable tasks. Priming for central venous access with the adult DIVA cognitive aid would involve retrieving the central venous access equipment to the bedside early during cannulation attempts, opening the equipment upon reaching the third lifeline, and performing emergency access after the final completed best attempt at the final lifeline. An earlier and more rapid transition through priming steps would be warranted for emergency or deteriorating clinical situations.

Central venous access will usually be the most appropriate vascular access option after all lifelines are exhausted. Central venous access is a safe and effective means of securing vascular access, albeit with an attendant risk of complications such as arterial puncture, haemorrhage/haematoma, pneumothorax/haemothorax (for jugular and subclavian access) and venous air embolism [22,23]. Routine use of ultrasound guidance is recommended for central venous access (level A evidence), as it significantly reduces complications, improves success, and minimises costs [22]. In a cardiac arrest scenario, IO access may, on occasion, be required. The International Liaison Committee on Resuscitation (ILCOR) recommends IO access if IV access is difficult or impossible in resuscitation scenarios [55]. IO access is faster than central venous access in a true emergency [23]. However, ILCOR notes that outcomes are likely to be better when drugs are administered IV verses IO [55]. Ultimately, the speed of transition through priming steps, the clinical need to bypass peripheral venous access attempts altogether and perform central venous access, the site of central access (femoral, internal jugular, or subclavian) or the need for IO access, will be context- and operator-specific. Importantly, in time-critical emergencies, an early recourse to context-appropriate emergency access should be taken for urgent resuscitation in all circumstances where peripheral IV access is not immediately possible.

## Discussion

The adult DIVA cognitive aid is an educational resource and proposed cognitive aid designed to assist during DIVA. The primary advantage of the adult DIVA cognitive aid is that it is a user-friendly resource that has been developed around evidence-based ultrasound and Seldinger-based techniques for peripheral venous access and is intended to assist practitioners across a broad range of technical ability-from beginner to advanced, and in a range of clinical environments. It is most suitable for anaesthesia and critical care practitioners adequately trained in real-time ultrasound-guided vascular access and Seldinger-based techniques but could be utilised by any healthcare provider who regularly encounters DIVA in their clinical practice if adequate supervised training in these techniques has been undertaken beforehand.

An additional advantage of the adult DIVA cognitive aid is that it is suitable for use during both elective and emergency scenarios. In an elective scenario, it is anticipated that the natural progression would be to move systematically through the direct puncture, ultrasound, and Seldinger-based lifelines. Once all lifelines are exhausted, in an emergency, or if the patient's clinical situation deteriorates, the practitioner is prompted to perform central venous access. For DIVA in the context of cardiac arrest, where peripheral or central access is not possible or appropriate, IO access is prompted. Of course, a true time-critical emergency obviates the need for cognitive aid, and practitioners should not hesitate to perform central venous or IO access in these situations if immediate peripheral IV access is not forthcoming, as recommended in international guidelines [22,23,55].

A unique aspect of the adult DIVA cognitive aid is that it brings direct puncture and ultrasound- and Seldinger-based techniques together in a convenient protocol. Previous guidelines for DIVA have focused heavily on direct puncture and ultrasound [16]. While much has been written on Seldinger techniques for vascular access in general [22,24,27,44-53], previous protocols have not assimilated evidence-based Seldinger techniques for DIVA. Indeed, there is a growing armamentarium of techniques now published, such as those included in Figure 1. The adult DIVA cognitive aid aims to raise awareness and nudge users towards the use of these readily available techniques.

An additional benefit of the adult DIVA cognitive aid is that it has been modelled on the Vortex airway approach and formulated with the behavioural psychology concept of choice architecture in mind. Its similarities with the Vortex approach is intended to engender a sense of cognitive ease and familiarity, while choice architecture, where all options are presented equally and no choices are forbidden or excluded, provides a flexible, user-friendly, and non-confrontational framework that is likely to be appreciated by clinicians of different levels of experience. Although unproven, it is hoped that by providing choice architecture, the adult DIVA aid may help reduce cognitive biases, shortcut decision-making, and operator stress, and increase adherence to evidence-based medicine, such as the use of early ultrasound guidance when DIVA is encountered or expected.

The adult DIVA cognitive aid emphasises a limit of two attempts for each lifeline in keeping with the Vortex approach thematic and this is also consistent with DIVA literature. It is recognised that repeated attempts at vascular access increase complication rates, cause significant patient distress, and treatment delays [2,22]. In an elective or non-time critical emergency, at least one attempt made by another experienced practitioner should be considered wherever practical prior to exiting the Green Zone and attempting emergency access solutions. The important point is that lifeline attempts are limited to maximise subsequent success, reduce harm, and avoid fixation errors.

The concept of mental imprinting is often used in marketing psychology and is aimed at increasing awareness and recall. The visual shape and colour of the adult DIVA cognitive aid alludes to a traffic ‘give way’ sign. This is intended to promote situational awareness and a ‘stop-and-think’ moment at each lifeline. The stop-and-think concept is a familiar concept in crisis management algorithms [12,54]. Central themes such as ’The Green Zone’, ‘two attempts’, and ‘lifelines’, akin to the Vortex approach, are also intended to harness the benefits of mental imprinting.

There are several limitations of the proposed DIVA cognitive aid. Firstly, while based upon evidence-based principles for DIVA, the adult DIVA cognitive aid itself remains an untested thesis and requires further investigation before it can be recommended for routine practice. As such, further studies are now required to assess efficacy endpoints such as success rates, time to cannulation, reductions in treatment delays, complication rates, and patient and staff satisfaction between the use and non-use of the adult DIVA cognitive aid or similar visual aids. Secondly, the level of evidence for the various techniques incorporated into the DIVA cognitive aid varies. Of the included techniques, the best evidence-base exists for ultrasound-guided cannulation, with level B evidence and a strong recommendation for ultrasound-guided cannulation when peripheral cannulation difficulty is anticipated in adults [22]. Since these consensus guidelines were published, a systematic review and meta-analysis of adequately powered randomised trials has added weight to this recommendation [21], with the caveat of moderate heterogeneity between trails. Regarding Seldinger-based techniques for DIVA, there is very limited high-quality evidence, with most of the evidence base derived from cohort studies, case series, and technical descriptions of novel techniques. Notwithstanding, all Seldinger-based peripheral venous access techniques incorporated into the adult DIVA cognitive aid have been used in clinical practice, with good success-rates. Furthermore, the Seldinger technique is a well-established and accepted standard in vascular access [22,23,56]. Thirdly, the adult DIVA cognitive aid may be more suitable for elective and non-time critical emergencies. While the central zone clearly provisions for emergency central venous or IO access during a time-critical emergency, a true time critical emergency such as a cardiac arrest would obviate the need for a cognitive aid altogether. In this type of emergency, the use of a cognitive aid could potentially cause harm, by distract staff and wasting precious time with the use of more complicated cannulation techniques and equipment. In these situations, practitioners should not hesitate to perform emergency access without delay. Fourthly, two attempts at each lifeline were chosen to provide consistency with the Vortex approach but is ultimately arbitrary. In controlled studies, two attempts at each lifeline could prove to be too many and cause significant patient discomfort, complication rates, and treatments delays. The optimal number of attempts with each technique or lifeline remains to be tested. Given the potential for significant patient discomfort and up to six cannulation attempts, local anaesthetic skin infiltration with a 25-Ga (or smaller) insulin syringe is imperative, as has been shown to reduce pain for most cannula sizes [13]. Fourthly, previous studies have noted that well-designed cognitive aids should contain visual information proportional and appropriate to the task being carried out [57]. For example, cognitive aids designed for time-critical emergencies, such as can’t intubate, can’t oxygenate (CICO) events, should not distract from airway management or be content-heavy. Conversely, aids for lower-acuity scenarios such as DIVA may provide more information or instruction. Notwithstanding, a limitation encountered during conception of the adult DIVA cognitive aid was the requirement for limited written content if the aid was to be user-friendly. Early iterations were information heavy, and this reduced clarity. Ultrasound and Seldinger techniques are moderately complex, and numerous potential optimisations were available for each lifeline. To address this issue, only prompts considered essential were included in Figure 1. In addition, supplementary cognitive aids (Figures 2, 3) were created to provide greater detail for evidence-based ultrasound-guided and ultrasound-assisted techniques, when required. Finally, while based on the Vortex model, the shape and design are arbitrary, and may not appeal to all users. Indeed, a linear chart, or an alternative flow diagram are equally plausible, provided it is based upon sound evidence-based techniques for DIVA. Similarly, some techniques, optimisations, and adjuncts were excluded due to weak evidence of efficacy, limited availability (e.g., advanced ultrasound technologies such as laser needle guides and 3D/4D ultrasound), and developer discretion, and may not match the ideals of other practitioners. Further, while a central aim when developing the adult DIVA cognitive aid was to include the most up-to-date and clinically useful techniques, additional modern evidence-based and clinically useful techniques may have been omitted or since emerged. Ultimately, the adult DIVA cognitive aid as presented here represents a prototype, or example of the techniques upon which a similar cognitive aid could be developed at departmental or institutional level, as well as to raise awareness of the cognitive biases that can negatively influence decision making.

## Conclusions

DIVA is a common problem with the potential to cause significant treatment delays and patient discomfort. The adult DIVA cognitive aid presented here is an educational resource and proposed cognitive aid designed to assist during DIVA. It is a user-friendly resource that has been formulated around evidence-based ultrasound and Seldinger techniques for peripheral venous access and is intended to assist practitioners across a broad range of technical abilities and in a range of clinical environments. Its similarity to the Vortex approach is intended to evoke familiarity and a sense of cognitive ease, while a choice-architecture-focused design provides a flexible scaffolding upon which clinical decisions can be made. With the wide-spread adoption of cognitive aids in modern hospital-based medical practice, the need for a DIVA aid now seems intuitive. Accordingly, it is hoped that hospital departments may benefit from using this DIVA cognitive aid as a teaching resource or cognitive aid when DIVA is encountered. Alternatively, departments may choose to use this model as a prototype to develop their own cognitive aids, tailored for individual departmental needs. Where DIVA cognitive aids are adopted, quality assurance and audit activities will be required to evaluate their implementation, utility, and safety. Studies are now required to compare cannulation success rates, time to cannulation, complication rates, and patient and staff satisfaction between the use and non-use of this DIVA cognitive aid.
